# Genetic mutation in *Escherichia coli* genome during adaptation to the murine intestine is optimized for the host diet

**DOI:** 10.1128/msystems.01123-23

**Published:** 2024-01-11

**Authors:** Tomoya Tsukimi, Nozomu Obana, Suguru Shigemori, Kazuharu Arakawa, Eiji Miyauchi, Jiayue Yang, Isaiah Song, Yujin Ashino, Masataka Wakayama, Tomoyoshi Soga, Masaru Tomita, Hiroshi Ohno, Hirotada Mori, Shinji Fukuda

**Affiliations:** 1 Institute for Advanced Biosciences, Keio University, Tsuruoka, Japan; 2 Systems Biology Program, Graduate School of Media and Governance, Keio University, Fujisawa, Japan; 3 Transborder Medical Research Center, Institute of Medicine, University of Tsukuba, Tsukuba, Japan; 4 Faculty of Environment and Information Studies, Keio University, Fujisawa, Japan; 5 RIKEN Center for Integrative Medical Sciences, Yokohama, Japan; 6 Institute for Molecular and Cellular Regulation, Gunma University, Maebashi, Japan; 7 Graduate School of Biological Science, Nara Institute of Science and Technology, Ikoma, Japan; 8 Institute of Animal Sciences, Guangdong Academy of Agricultural Sciences, Guangzhou, Guangdong, China; 9 Gut Environmental Design Group, Kanagawa Institute of Industrial Science and Technology, Kawasaki, Japan; 10 Laboratory for Regenerative Microbiology, Juntendo University Graduate School of Medicine, Tokyo, Japan; The University of Maine, Orono, Maine, USA

**Keywords:** gut microbiota, *Escherichia coli*, genetic mutation, intestinal colonization, intestinal nutrient

## Abstract

Mammalian gut microbes colonize the intestinal tract of their host and adapt to establish a microbial ecosystem. The host diet changes the nutrient profile of the intestine and has a high impact on microbiota composition. Genetic mutations in *Escherichia coli*, a prevalent species in the human gut, allow for adaptation to the mammalian intestine, as reported in previous studies. However, the extent of colonization fitness in the intestine elevated by genetic mutation and the effects of diet change on these mutations in *E. coli* are still poorly known. Here, we show that notable mutations in sugar metabolism-related genes (*gatC*, *araC*, and *malI*) were detected in the *E. coli* K-12 genome just 2 weeks after colonization in the germ-free mouse intestine. In addition to elevated fitness by deletion of *gatC*, as previously reported, deletion of *araC* and *malI* also elevated *E. coli* fitness in the murine intestine in a host diet-dependent manner. *In vitro* cultures of medium containing nutrients abundant in the intestine (e.g., galactose, N-acetylglucosamine, and asparagine) also showed increased *E. coli* fitness after deletion of the genes-of-interest associated with their metabolism. Furthermore, the host diet was found to influence the developmental trajectory of gene mutations in *E. coli*. Taken together, we suggest that genetic mutations in *E. coli* are selected in response to the intestinal environment, which facilitates efficient utilization of nutrients abundant in the intestine under laboratory conditions. Our study offers some insight into the possible adaptation mechanisms of gut microbes.

**IMPORTANCE:**

The gut microbiota is closely associated with human health and is greatly impacted by the host diet. Bacteria such as *Escherichia coli* live in the gut all throughout the life of a human host and adapt to the intestinal environment. Adaptive mutations in *E. coli* are reported to enhance fitness in the mammalian intestine, but to what extent is still poorly known. It is also unknown whether the host diet affects what genes are mutated and to what extent fitness is affected. This study suggests that genetic mutations in the *E. coli* K-12 strain are selected in response to the intestinal environment and facilitate efficient utilization of abundant nutrients in the germ-free mouse intestine. Our study provides a better understanding of these intestinal adaptation mechanisms of gut microbes.

## INTRODUCTION

The human gut harbors as many gut microbes as host cells (1). The total number of genes carried by the gut microbiota is estimated to be greater than the number of host genes (2, 3). The gut microbiota creates a complex ecosystem through interactions between bacteria and host cells. Although the host genetic background influences the composition of the gut microbiota (4), cohort data indicate that environmental factors, including diet, have a significantly greater impact (5). Microbiota-accessible carbohydrates (MACs) are found in host dietary fibers, and they are the primary nutrients utilized by the gut microbiota. MACs greatly influence the gut microbiota composition (6–8). Long-term changes from high-MAC diets to low-MAC diets result in the irreversible loss of certain species (9). Dietary composition exerts selective pressure for not only species but also variants and substrains within the same species (10, 11), suggesting that genetic mutations in the gut bacterial genome occur during adaptation to the gut environment. However, these studies have mainly focused on the relative abundance of species or bacterial genes and information on the adaptation process itself, including the selection of advantageous mutations, is lacking.

*Escherichia coli*, which lives in the human gut throughout the life of a human host (12–14), is widely used for experimental adaptation studies due to the ease of genetic engineering. Recently, experiments using *E. coli-*colonized mice have uncovered several adaptive mutations in the *E. coli* genome (15–18). The functions of the mutated genes identified were mainly involved in bacterial metabolism, for example, *gat* genes involved in galactitol metabolism (16), *lrp* encoding a global transcriptional regulator of amino acid metabolism (17), and *dgoR* encoding a repressor of galactonate metabolism genes (18). Furthermore, mutated genes were altered by co-colonization with *Blautia coccoides* through modulation of the gut metabolome (17). These findings suggest that the genetic mutation of *E. coli* reflects the intestinal metabolome environment, where various metabolites are present. In addition to the existence of other bacterial species, the host diet can also alter the gut environment. Indeed, the genetic signature of *Bacteroides thetaiotaomicron* during colonization of the murine intestine is diet specific, and daily switching of diet increases genetic diversity (19). Metagenomic fragments collected from mice inoculated with probiotic *E. coli* Nissle 1917 showed that certain gene functions were enriched depending on host dietary condition (15), suggesting that diet affects genetic mutation within the *E. coli* genome during adaptation. However, the extent to which dietary changes affect the fitness of such mutations in *E. coli* and whether the changes alter the mutational landscape of *E. coli* colonization in the murine intestine are still poorly known. Therefore, we focused on genetic mutations during intestinal colonization in this study.

To investigate the relationship between the host’s gut environment and mutations, we used an *E. coli* hyper-mutator strain (Δ*mutL* and Δ*mutS*), which is derived from *E. coli* K-12 strain, and germ-free (GF) mice. Hyper-mutator strains are more likely to accumulate mutations in their genomes, and GF mice enable us to clearly detect mutations of these strains within the mammalian intestine without the interference of other variables such as other resident bacteria. This framework streamlined our investigation on the evolution of highly adaptive *E. coli* strains within the host gut environment. Through the study results, we were able to confirm that the deletion of several genes commonly mutated in independent experiments elevated the fitness of *E. coli* in the murine intestine, and we observed that the extent of fitness depended on the host diet. Furthermore, we found that in an *in vitro* setting, these gene deletions enhanced the ability to utilize specific nutrients that was abundant in the intestinal lumen of mice depending on their diet.

## RESULTS

### Screening of mutated genes in *E. coli* during murine intestinal colonization

**Fig 1:**
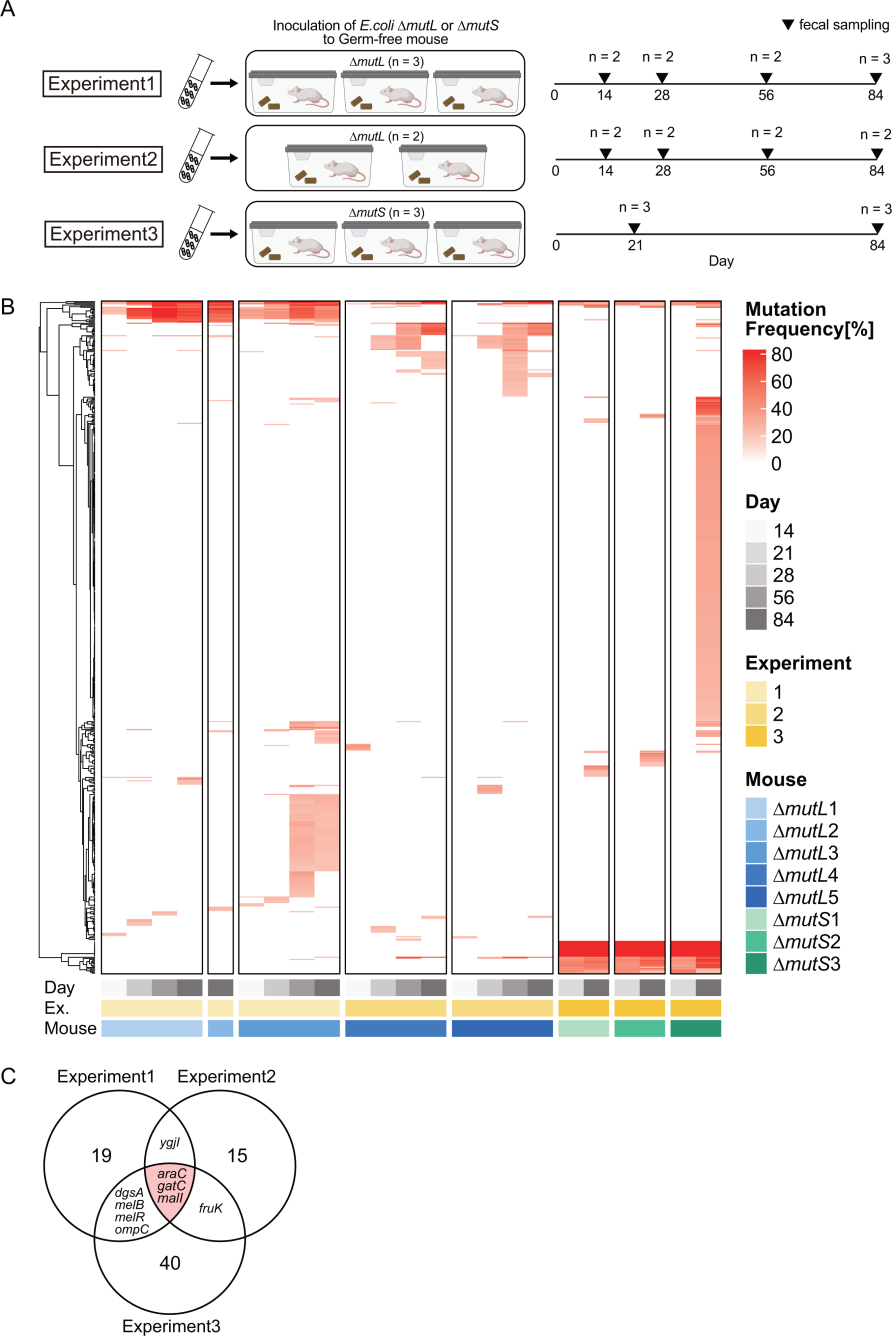
Mutated genes in hyper-mutator *E. coli* during adaptation to the murine intestinal environment. (**A**) Schematic representation of the mutated gene screening experiment. (**B**) Mutated genes in *E. coli* during intestinal colonization in three independent experiments. Each column on the heatmap indicates a sampling timepoint of a mouse, whereas each row indicates a mutated gene that was estimated to have “HIGH” or ”MODERATE” effect on protein function by SnpEff (25). Estimates derived from mutations at different positions within the same gene were consolidated. The dendrogram on the left was calculated by complete clustering of Euclidean distance. The boxes on the bottom represent the sampling points of feces, experiments, and mouse replicates. The first experiment using Δ*mutL*: *n* = 3; second: *n* = 2; and Δ*mutS*: *n* = 3. Heatmap color indicates the frequency of the mutation in each mouse. (**C**) Venn diagram of commonly mutated genes in two or more mice in each experiment on day 84.

We investigated the adaptation of *Escherichia coli* to the mouse gut by colonizing GF mice with an *Escherichia coli* K-12 hyper-mutator strain (Δ*mutL* or Δ*mutS*) (20, 21). The *mut* genes encode a mismatch repair (MMR) protein that normally prevents genetic aberrations in the genome. The mutation frequencies in the Δ*mutL* and Δ*mutS* strains are more than 100-fold higher than in the wild-type (22, 23). *In vitro* studies have shown that mutator-specific mutations are very few relative to all mutations (24). We inoculated the hyper-mutator strains into three groups of GF mice to detect commonly mutated genes in independent experiments (Fig. 1A). Rapid gene mutations were observed, and the mutation trajectories of *E. coli* within the same experiment tended to be similar (Fig. 1B).

We identified the mutations that likely affected protein function (e.g., non-synonymous coding, frameshift, and stop gain mutations; see Materials and Methods) from the day 84 samples and found that nine gene mutations were commonly detected in two or more experiments (Fig. 1C). Mutations in *gatC*, *araC*, and *malI* genes were observed in most mice at day 14, and all mice at day 84 (Fig. S1). Frameshift and stop codon acquisition mutations were observed in these three genes (Table 1), suggesting that their loss of function contributed to intestinal colonization. These genes encode a galactitol transporter (26), transcriptional factors for the arabinose operon (27, 28), and that for the maltose operon (29), respectively (Table 1). Mutations in the *gat* operon—which is responsible for galactitol metabolism—in *E. coli* strain K-12 have been reported to occur in the mouse intestine in previous studies (16, 17, 30). The *gat* operon is constitutively expressed in *E. coli* K-12 because the *gat* operon repressor gene, *gatR*, is dysfunctional due to an insertion sequence (IS) (16, 31). In variants of this strain in which the *gatR* pseudogene has been replaced by a functional version, inactivation of *gatR* by IS has been observed to occur during intestinal colonization in mice (31). However, the growth of wild-type *E. coli* K-12, in which the expression of *gat* operon genes was enhanced, was inhibited by galactitol *in vitro*, while the *gat* gene-mutated strain was not (16). Because the hyper-mutator strains used in our study are derived from *E. coli* K-12, which possess a dysfunctional *gatR* gene due to the insertion sequence, we considered that the accumulation of the *gatC* mutation might be a consequence of constitutive *gat* operon expression in *E. coli* K-12. Thus, we shifted our focus on the other gene mutations in accordance with our original goal of investigating the adaptation to the intestinal environment. In regard to *malI*, a previous study observed *malI* mutations in some *E. coli* K-12-inoculated mice. However, mutations in this gene were not prevalently detected in all mice used in the study (32).

**TABLE 1:** Commonly mutated genes in three experiments on day 84

Gene	Function	Position	Ref.	Alt.	Mutation frequency (%)*^a^*	Mutation type
*araC*	Arabinose operon regulatory protein	70424	A	G	66.40	Non-synonymous coding
		70484	A	AT	24.92	Frame shift
		70517	G	A	35.06	Non-synonymous coding
		70547	AG	A	22.21	Frame shift
		70698	G	A	25.00	Stop gained
*dgsA*	DNA-binding transcriptional repressor Mlc	1665754	G	A	36.02	Stop gained
		1665849	T	C	56.61	Non-synonymous coding
		1665912	G	GC	49.54	Frame shift
		1666162	G	GT	44.79	Frame shift
		1666357	GC	G	28.49	Frame shift
*fruK*	1-Phosphofructokinase	2259847	G	A	47.99	Non-synonymous coding
		2260075	C	T	57.38	Non-synonymous coding
*gatC*	PTS system galactitol-specific EIIC component	2171258	A	G	23.12	Non-synonymous coding
		2171319	G	A	62.73	Stop gained
		2171472	G	A	64.39	Stop gained
		2171604	GC	G	56.58	Frame shift
		2171775	CT	C	26.86	Frame shift
*malI*	Maltose regulon regulatory protein	1696432	GC	G	26.46	Frame shift
		1696596	G	GC	56.32	Frame shift
		1696908	C	CA	21.28	Frame shift
		1696908	CA	C	61.33	Frame shift
		1697167	G	A	22.75	Non-synonymous coding
*melB*	Melibiose:H+/Na+/Li+symporter	4341572	A	G	23.49	Non-synonymous coding
		4341740	G	A	31.15	Non-synonymous coding
		4342227	A	G	26.33	Non-synonymous coding
		4342446	T	C	52.61	Non-synonymous coding
		4342508	A	G	26.17	Non-synonymous coding
*melR*	Melibiose operon regulatory protein	4338978	A	G	27.68	Non-synonymous coding
		4339297	C	T	50.62	Non-synonymous coding
		4339530	C	T	21.30	Non-synonymous coding
*ompC*	Outer membrane porin C	2310395	T	C	53.25	Non-synonymous coding
*ygjI*	Inner membrane transporter	3224549	C	CT	28.64	Frame shift
		3224687	C	CG	52.62	Frame shift
		3224687	CG	C	21.39	Frame shift

^*a*^ Mean frequency of mutation in *E. coli* within all mice that harbored this mutation on day 84.

### The mutated genes contribute to increased colonization fitness in the mouse intestine

**Fig 2:**
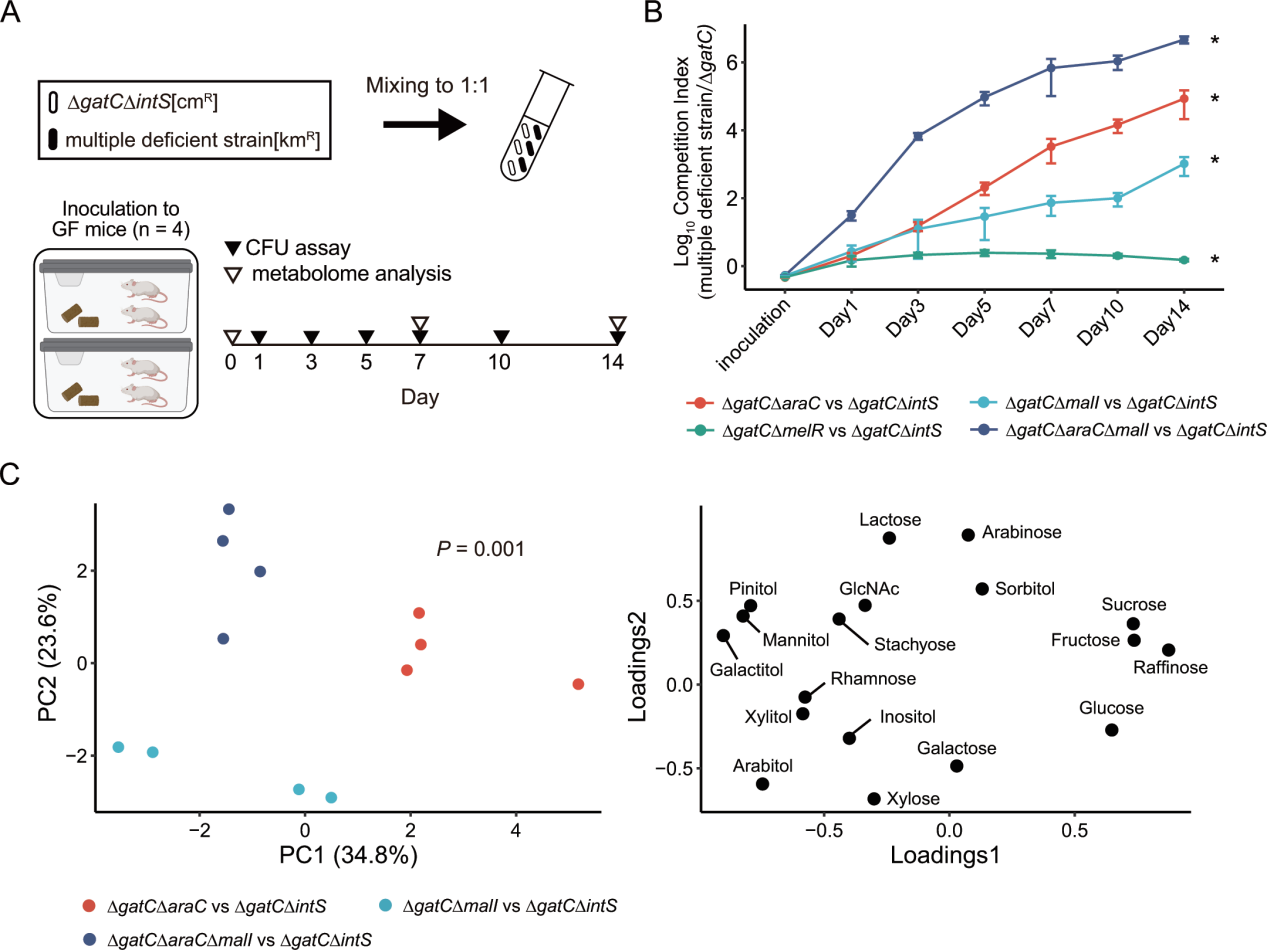
Deletion of mutated genes confers fitness to *E. coli* in the murine intestine. (**A**) Schematic representation of *in vivo* competition assay between Δ*gatC* and double- or triple-mutant strains. (**B**) *In vivo* competition assay between Δ*gatC* and multiple-mutant strains (*n* = 4). The log10 competition index (CFU of double- or triple-mutants divided by that of Δ*gatC*) is shown as the mean  ±  SEM. *P  <  0.05 (paired *t* test with Holm’s correction between Δ*gatC* and each double or triple mutant on day 14). (**C**) Principal component analysis (PCA) score plot of sugar concentration in the feces sampled on day 14 (left panel) and its loadings (right panel). In the PCA panel, each point represents a sample, and the color indicates inoculated strains. *P* value was calculated with PERMANOVA.

To investigate the contributions of these gene mutations on fitness in the mouse intestine, we constructed multiple-mutant strains. Here, we focused on the *araC* and *malI* genes, whose mutations were commonly detected in all mice. Competitive fitness of *araC* and *malI* mutants was compared with Δ*gatC*, as the *gat* gene mutation is quickly introduced during murine intestinal colonization, is eventually acquired at a consistently high frequency, and appears to have the great impact on fitness (16). As such, we introduced this highly dominant mutation to our strains as a baseline to diminish its influence on our results and compare it with other mutations. Double mutants (Δ*gatC*Δ*araC*, Δ*gatC*Δ*malI*, and Δ*gatC*Δ*melR*) and a triple mutant (Δ*gatC*Δ*araC*Δ*malI*) were co-inoculated with a Δ*gatC* single-mutant in GF mice to compare their ability to establish intestinal colonization (Fig. 2A). We used Δ*gatC*Δ*melR* as the baseline for comparison of Δ*gatC*Δ*araC* and Δ*gatC*Δ*malI* since the mutation in *melR* was detected in only two groups during colonization of the hyper-mutator strain, which suggests that any fitness conferred by *melR* mutation is assumed to be less than that of *araC* or *malI* mutation. In addition, *melR*, which encodes a transcription factor for the melibiose operon, shares a common feature with *araC* and *malI* in that it also encodes a sugar metabolism-related transcription factor (Fig. 1C).

By fourteen days after inoculation, Δ*gatC*Δ*araC*Δ*malI*, Δ*gatC*Δ*araC*, and Δ*gatC*Δ*malI* significantly outcompeted Δ*gatC* by a >1,000-fold increase in CFU (Fig. 2B). Δ*gatC*Δ*melR* also outcompeted Δ*gatC*, but the CFU of Δ*gatC*Δ*melR* was just 1.5-fold greater than that of Δ*gatC*. These results suggest that the loss of function of *araC* and *malI* enhances colonization fitness in the murine intestine for *E. coli*. The competition index was highest in the order of Δ*gatC*Δ*araC*Δ*malI*, Δ*gatC*Δ*araC*, and Δ*gatC*Δ*malI*. This suggests that *araC*-deficiency confers a greater fitness increase to *E. coli* than *malI* deficiency, and deficiency in both genes additively contributes to colonization fitness. Given that the mutations in *araC*, *gatC*, and *malI* were detected in most of the mice on day 14 (Fig. S1), mutations resulting in highly elevated fitness would be observed earlier during colonization than those with lesser or negligible impact.

Since *araC* and *malI* are related to sugar utilization, we measured the amounts of sugars in the *E. coli* mutant-colonized mouse intestine to identify possible patterns that would implicate sugar metabolism with intestinal fitness. Galactitol was detected in mouse cecal contents, colon contents, and feces (Fig. S2A), suggesting that the occurrence of *gatC* mutations (Fig. 1C) was to avoid galactitol metabolism because galactitol metabolism is known to severely inhibit growth for unknown reasons (16). Principal component analysis (PCA) score plots of sugar profiles based on the fecal concentrations of 25 different sugars were distinct in each mouse group, implying that each gene mutation differentially influences the sugar utilization patterns of *E. coli* in the murine intestine (Fig. 2C). Therefore, we hypothesized that altering sugar metabolism in *E. coli* might be a key to obtaining greater fitness.

### The mutated genes are optimized and selected for nutrient composition in the intestine, which changes with the host diet

**Fig 3:**
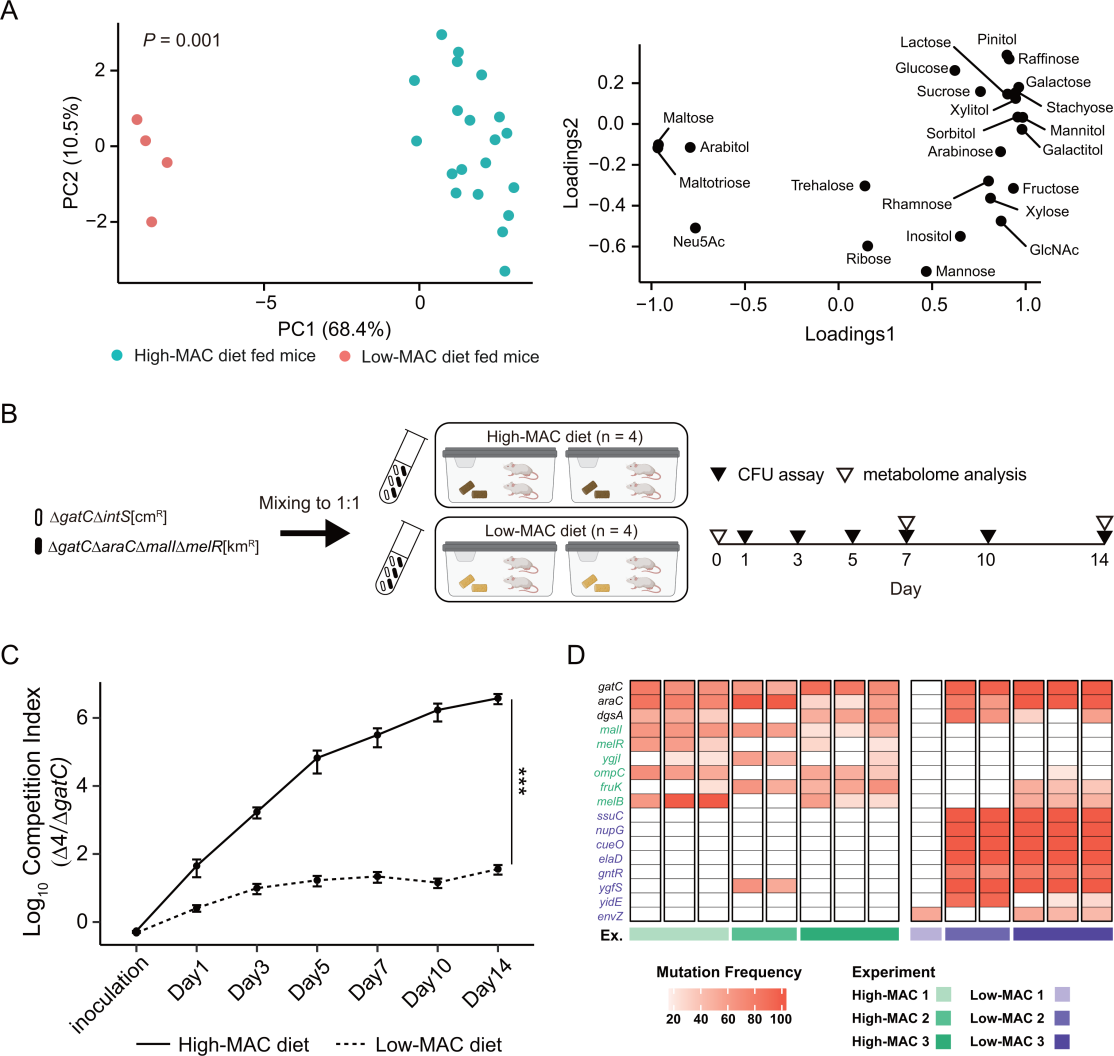
Trajectory of advantageous mutations depends on nutrient composition in the intestine. (**A**) Principal component analysis of the amounts of fecal sugars before inoculation (left panel) and its loading plot (right panel). In the PCA panel, each point represents a sample, and the color indicates the given diet. (**B**) Schematic representation of *in vivo* competition assay between Δ*gatC*Δ*araC*Δ*malI*Δ*melR* and Δ*gatC* and in different diet-fed mice. (**C**) *In vivo* competition assay between Δ*gatC*Δ*araC*Δ*malI*Δ*melR* and Δ*gatC* in different diet-fed mice. The log10 competition index (CFU of quadruple-mutant strain divided that of Δ*gatC*) is shown as the mean  ±  SEM (*n* = 4). The line pattern represents diet (high-MAC diet, solid; low-MAC diet, dashed). ***P  <  0.005 (two-way ANOVA). (**D**) Commonly mutated genes detected in more than half of high-MAC diet-fed mice (*n* = 8) or low-MAC diet-fed mice (*n* = 6) on day 84. Each column on the heatmap indicates a mouse individual, whereas each row indicates a mutated gene that was predicted to affect protein function. The frequencies of mutations at different positions within the same gene are consolidated. The boxes on the bottom denote the experiment IDs. Mutated genes detected in more than half of high-MAC diet-fed mice, low-MAC diet-fed mice, or both groups are written in green, purple, and black, respectively.

Host diet, especially MACs, heavily influences the profile of intestinal carbohydrates, which the gut microbiota can utilize, and subsequently affects gut microbiota composition (6–9). We reasoned that if gene mutation-induced changes in sugar metabolism by *E. coli* enhance fitness in the intestine, dietary changes that affect sugar and other carbohydrate levels may lead to different results in competition between *E. coli* strains. We confirmed that intestinal sugar profiles in GF mice fed with different diets (high-MAC diet, used in mutated gene screening [Fig. 1A] and *in vivo* competition assays [Fig. 2A], and low-MAC diet [Table S1]) were significantly different (Fig. 3A). We co-inoculated two groups of GF mice that were fed these diets with Δ*gatC*Δ*araC*Δ*malI*Δ*melR* and Δ*gatC*. In both groups, Δ*gatC*Δ*araC*Δ*malI*Δ*melR* outcompeted Δ*gatC*. However, the competition index in the low-MAC diet group was 100,000 times smaller than that of the high-MAC diet group (Fig. 3B and C). The significant differences in sugar composition and quantity in feces suggest that certain carbohydrates might be involved in the fitness of the multiple-mutant strain. (Fig. 3A; Fig. S2B).

To determine whether host dietary changes affect the mutational trajectory, we inoculated Δ*mutL* in GF mice fed a low-MAC diet and monitored mutations in the *E. coli* genome. We observed that nine genes in the high-MAC diet group and 11 genes in the low-MAC diet group were mutated in more than half of the mice in each group (Fig. 3D; Table S2; see Materials and Methods). Although *gatC*, *araC*, and *dgsA* mutations were found in both groups, the other genes were found to be mutated in >50% of mice in their respective diet group and mutations were largely unique to host diet. One mouse in the low-MAC diet group (low-MAC 1) displayed a different pattern of mutated genes from the other mice. Although *gatC* mutation was not detected in this mouse, the *gatB* gene, which encodes for a galactitol transporter subunit along with *gatC*, was mutated. Therefore, the *E. coli* in the mouse seemed to avoid growth inhibition by galactitol metabolism by selecting for this mutation in a similar fashion as *gatC*-mutated strains in other mice. Although some mutated genes were detected in more than half of mice in both diets, the results suggested that the mutated gene was selected for nutrient composition in the intestinal lumen, which changes with the host diet.

### The host diet alters the expression of genes in *E. coli* responsible for the metabolism of nutrients abundant in the intestine

The genes *araC* and *malI*, whose deletions conferred colonization fitness to *E. coli* in the high-MAC diet-fed mice, encode transcription factors. AraC activates the *ara* operon involved in arabinose metabolism in the presence of arabinose (27), the amount of which was higher in the high-MAC group than in the low-MAC group (Fig. S2B). Notably, the *E. coli* K-12 BW25113 strain we used cannot utilize arabinose as a carbon source because of the deletion of the *araBAD* gene. To determine whether arabinose is responsible for the advantage of *araC* deficiency, we cocultured Δ*gatC* and Δ*gatC*Δ*araC* in M9 + glucose minimal media with or without arabinose. As a result, although *araC* deficiency indeed increased fitness in the presence of arabinose (Fig. S3), the extent of increased fitness was smaller than that observed *in vivo* in terms of competition index (Fig. 2B). Considering how our *E. coli* strain should be unable to metabolize arabinose, *araC* deficiency in this strain may prevent transcription and translation of the *ara* genes in the presence of arabinose, which leads cells to use the energy to grow more efficiently. In addition, recent studies have shown that AraC regulates a wide range of gene expression (33), which suggests the involvement of other metabolic processes. MalI is a repressor of the *malXY* operon encoding maltose transporters (29), and the deletion of *malI* is expected to enhance maltose uptake and dissimilation. However, maltose was not detected in the feces of the high-MAC diet group prior to inoculation, suggesting that it was not readily available in the intestinal environment (Fig. S2B). Therefore, we speculated that colonization fitness in the high-MAC diet conferred by *araC* and *malI* deletion was due to other metabolites.

**Fig 4:**
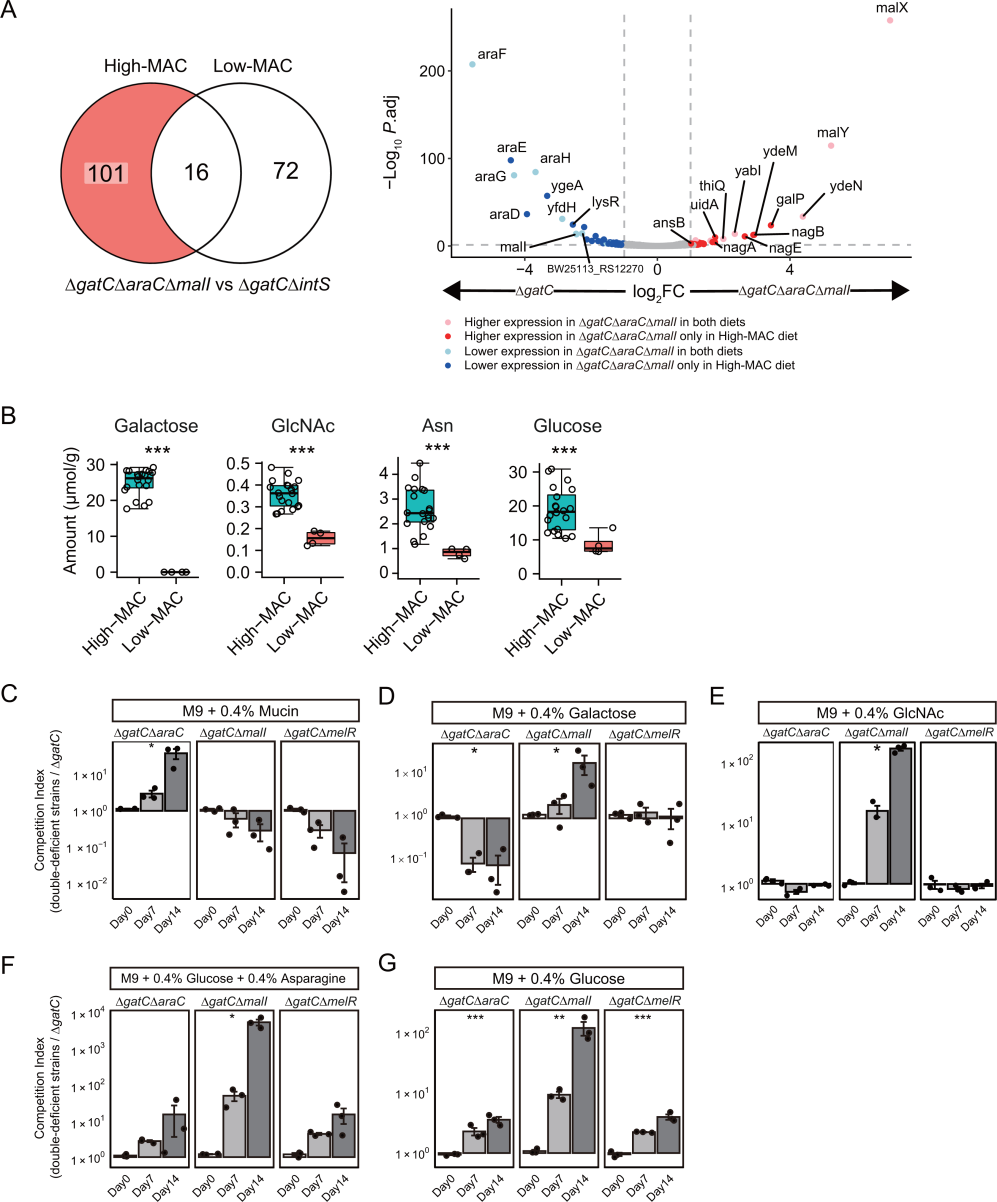
*AraC* and *malI* deficiency enhances fitness in media containing the corresponding nutrients among those abundant in the intestines of high-MAC diet-fed mice. (**A**) A comparison of DEGs between Δ*gatC*Δ*araC*Δ*malI* and Δ*gatC* in high-MAC or low-MAC diet-fed mice. DEGs between the strains were assessed using DESeq2 (42). Venn diagram (left panel) shows the number of DEGs (FDR < 0.05 and log2 fold change > 1 or < −1) between Δ*gatC*Δ*araC*Δ*malI* and Δ*gatC* in high-MAC or low-MAC diet-fed mice (*n* = 3). The volcano plot (right panel) shows the *P* value and log2 fold changes of gene expression comparing Δ*gatC*Δ*araC*Δ*malI* and Δ*gatC* in high-MAC diet-fed mice. Transcripts with significant differential expression between the compared strains are highlighted according to designated color codes. (**B**) The amounts of metabolites in feces of high-MAC or low-MAC diet-fed mice before inoculation. ***P  <  0.005 (Welch’s *t* test with FDR correction). (**C through G**) *In vitro* competition assay between Δ*gatC* and double-mutant strains (*n* = 3) in M9 + 0.4% mucin (**C**), M9 + 0.4% galactose (**D**), M9 + 0.4% GlcNAc (**E**), M9 + 0.4% glucose + 0.4% asparagine (**F**), and M9 + 0.4% glucose (**G**). The log10 competition index (CFU of a double-mutant strain divided that of Δ*gatC*) is shown as the mean  ±  SEM. *P  <  0.05, **P  <  0.01, and ***P  <  0.005 (paired *t* test with Holm’s correction between Δ*gatC* and each double-mutant strain on day 14).

To elucidate the mechanisms of colonization fitness enhancement, we analyzed the influence of the deletion of *araC* and *malI* on gene expression in *E. coli* colonized in the intestine. We inoculated either Δ*gatC*Δ*araC*Δ*malI* or Δ*gatC* in GF mice fed a high-MAC or low-MAC diet and extracted total bacterial RNA. Differentially expressed genes (DEGs) between the strains differed according to the host diet (Fig. 4A). We hypothesized that DEGs detected only in high-MAC diet-fed mice (101 genes) might contribute to the enhanced fitness of Δ*gatC*Δ*araC*Δ*malI* in high-MAC diet-fed mice. The expression of *galP*, *nagABE*, and *ydeM* was upregulated in Δ*gatC*Δ*araC*Δ*malI* in the high-MAC diet-fed mice compared with Δ*gatC* (Fig. 4A; Table S3). *galP* encodes a galactose transporter (34), and *nagABE* is involved in the metabolism of N-acetylglucosamine (GlcNAc) (35, 36), a component of mucin. *ydeM* encodes an anaerobic sulfatase maturation enzyme (37) and is co-transcribed with *ydeN*, which encodes a sulfatase (38). *ydeN* is a direct target of AraC, and the Δ*araC* strain highly expresses *ydeMN* (33). Although the detailed substrates of YdeN have not been reported, YdeN of *E. coli* K-12 strain showed 33% amino acid sequence similarity with BT4656, which encodes N-acetylglucosamine-6-O-sulfatase from *Bacteroides thetaiotaomicron*. Sulfatases are required for *Bacteroides* to access mucosal glycans in the mammalian intestine (39). Therefore, we speculate that *ydeMN* is related to mucin metabolism by *E. coli*. Among asparagine metabolism-related genes, the expression of the synthesis gene (40) (*asnB*, log2FC = −0.98, *P* = 0.001) was decreased, and the expression of the degradation gene (41) (*ansB*, log2FC = 1.01, *P* = 0.047) was increased (Fig. 4A). These results prompted us to test whether these metabolites are responsible for the colonization fitness of Δ*gatC*Δ*araC*Δ*malI* in the high-MAC diet. Indeed, the galactose, GlcNAc, and asparagine concentrations were higher in the feces of the high-MAC diet group (Fig. 4B; Fig. S4A).

### The advantage of *araC* and *malI* mutations is reproducible *in vitro* when their corresponding nutrients, abundant in mice fed a high-MAC diet, are utilized as a sole carbon source

To determine whether these metabolites are responsible for the fitness advantage associated with *araC* and *malI* deficiency, we co-cultured Δ*gatC*Δ*araC*, Δ*gatC*Δ*malI*, or Δ*gatC*Δ*melR* with Δ*gatC* in M9-based minimal media containing mucin, galactose, GlcNAc, or asparagine as a carbon source. Because the strains were not able to grow in M9 + asparagine medium, we used glucose + asparagine medium. Although the amount of glucose was also higher in the feces of the high-MAC diet group than in the low-MAC diet group, the average amount of glucose in the low-MAC diet group was 8.7 µmol/g, which was higher than most other sugars in the low-MAC diet group (Fig. 4B; Fig. S2B).

By 14 days after inoculation, Δ*gatC*Δ*araC* outcompeted Δ*gatC* in mucin medium (Fig. 4C). Mucin, the main component of mucus, is a source of nutrients for some intestinal microbes (e.g., *B. thetaiotaomicron* and *Akkermansia muciniphila*) (43–45), and *E. coli* can also utilize mucin (46). Although the possibility that the commercial mucin (bound sialic acid 0.5 – 1.5%; free N-acetylneuraminic acid ≤ 0.2%) we used contained trace amounts of other growth-promoting compounds cannot be ruled out, the results suggest that the AraC regulon contains mucin degradation-related genes that allow for metabolism of mucin. Δ*gatC*Δ*malI* outcompeted Δ*gatC* in galactose, GlcNAc, glucose + asparagine, and glucose media (Fig. 4D through G). Although Δ*gatC*Δ*malI* outcompeted Δ*gatC* in glucose medium, the competition index was higher in glucose + asparagine medium, suggesting that asparagine conferred *in vitro* fitness to Δ*gatC*Δ*malI* (Fig. 4F and G). In summary, we predict that *araC* and *malI* deficiency enhances the metabolism of nutrients abundant in the mouse intestine, which results in increased intestinal colonization fitness. Interestingly, Δ*gatC*Δ*araC*Δ*malI* did not outcompete Δ*gatC* in the media containing mucin, galactose, and GlcNAc as a sole carbon source (Fig. S5A). However, the triple-mutant strain outcompeted Δ*gatC* in mucin + galactose medium. Furthermore, the competition index of Δ*gatC*Δ*araC*Δ*malI* was approximately threefold larger than that of Δ*gatC*Δ*araC* and Δ*gatC*Δ*malI* in the same medium (Fig. S5B).

GlcNAc, asparagine, and glucose were also present in the intestine of the low-MAC diet-fed mice (Fig. 4B), though at lower concentrations. Therefore, these metabolites might also contribute to the slight predominance of the quadruple-mutant strain in the low-MAC diet-fed mice (Fig. 3C).

## DISCUSSION

In this study, rapid mutation of sugar metabolism-related genes was observed in *E. coli* mutator strains (Fig. 1BC; Fig. S1). Deletion of these mutated genes conferred colonization fitness to an extent that increased based on how many mice a given gene was mutated in (Fig. 2B). The host diet affected the selection of mutated genes and their associated fitness increase (Fig. 3C and D). The deletion of the two most commonly mutated genes, *araC* and *malI*, changed the expression of other metabolism-related genes responsible for the utilization of metabolites that were abundant in the intestinal environment *in vivo* (Fig. 4AB) and enhanced *in vitro* fitness in media containing these metabolites as carbon sources (Fig. 4C through G; Fig. S5).

The feature of mutated genes was consistent with previous studies on the point that metabolism-related genes were mutated (16–19, 30), suggesting that genetic mutation of gut microbes strongly reflects the intestinal metabolome environment. The significant fitness impact of the deletion of *araC* and *malI* might be related to the fact that these genes encode transcription factors. In fact, AraC regulates the expression of a wide range of genes (33), which suggests the involvement of other metabolic processes. We expected *araC* deficiency to have a higher impact on fitness because *in vivo* competition assays showed that deletion of *araC* resulted in greater fitness in *E. coli* than *malI* (Fig. 2B). However, the enhanced fitness conferred by *araC* deficiency only seemed to be reflected in mucin and arabinose-supplemented media, and the extent of fitness tended to be lower than *malI* deficiency in our *in vitro* competition assay (Fig. 4C through G; Fig. S3). One possible implication of this is that other metabolites in the intestine contribute to the fitness-enhancing effects of *araC* inactivation. Any associations between *malI* and the metabolism of galactose, GlcNAc, and asparagine have not been reported. However, the DEGs between Δ*gatC*Δ*araC*Δ*malI* and Δ*gatC in vivo* and *in vitro* competition assays in our study suggest that MalI might regulate more metabolic processes (Fig. 4A and D through G). Some studies also reported that genetic mutations in *E. coli* during adaptation to the murine intestine promoted the metabolism of various metabolites through modulation of non-canonical regulatory targets. For example, a mutation in *lacI*, which encodes a transcription factor for the lactose operon, promoted raffinose metabolism, and a mutation in *gntT*, which encodes a gluconate transporter, promoted GlcNAc and mucin metabolism (15, 32). Interestingly, Δ*gatC*Δ*araC*Δ*malI* did not outcompete Δ*gatC* in mucin, galactose, and GlcNAc media *in vitro* but outcompeted in mucin + galactose medium (Fig. S5), suggesting that the gene mutations in *araC* and *malI* were selected simultaneously to optimize for the complex nutrient profile in the mammalian intestine.

The findings in this study are subject to two major limitations. The first is the use of GF mice. Indigenously colonized bacteria can affect the adaptation of invading bacteria through horizontal gene transfer (47, 48) and alteration of the metabolic environment of the gut (17). In particular, the existence of bacteria capable of metabolizing complex plant polysaccharides may greatly increase the carbon sources available to *E coli*. Therefore, mutation profiles and the extent of enhanced fitness by deletion of the commonly mutated genes, e.g., *gatC*, *araC*, and *malI*, might change with the existence of resident bacteria. It is important to acknowledge that this study focused on the relationships between mutated genes, their effected changes in gene expression, and metabolites in the intestine by excluding the variables introduced by other resident bacteria. The second is the use of *E. coli* K-12, which is a laboratory strain. *E. coli* K-12 is an attenuated strain, and it does not normally colonize the human intestinal tract (49). Both commensal and pathogenic *E. coli* strains inhabit the mammalian gut, and they exhibit diverse phenotypic and genotypic variants, including differences in carbon source utilization (50). These differences in *E. coli* strains should be considered when generalizing the results of this study.

Notwithstanding these limitations, we demonstrated that genetic mutations in *E. coli* are selected to optimize metabolism in response to the host diet-influenced gut environment. Recently, the gut microbiota has been targeted to prevent and treat diseases in humans. Some studies show attempts to manipulate it by using methods such as fecal microbiota transplantation (FMT) or probiotics (15, 51, 52). Our study showed that the metabolic profile, which can be altered by the host diet, has high impact on the colonization fitness of gut microbiota. This result suggests that the host diet might improve the above microbiota-targeting treatments. Overall, this study offers some insight into possible adaptation mechanism of the microbiota in the intestinal environment.

## MATERIALS AND METHODS

### Animals

The strain, genetic background, age, sex, replicates, and housing laboratory of the mice used in this study are listed in Table S4. The animals were bred and raised in axenic isolators under controlled light conditions (12-h light/12-h dark cycle). They were given sterilized water and fed CMF (Oriental Yeast Co. Ltd., Itabashi, Tokyo, Japan) as a high-MAC diet. Low-MAC diet-fed mice group changed their diet from CMF to AIN-93G (Oriental Yeast Co. Ltd., Itabashi, Tokyo, Japan) 1 week before the start of the experiment. Animal experiment procedures were approved by the Institutional Animal Care and Use Committee of the RIKEN Yokohama Branch and University of Tsukuba.

### Bacterial strains

The *Escherichia coli* K-12 BW38029 hyper-mutator strain (Δ*mutL*) was provided by professor Hirotada Mori at the Nara Institute of Science and Technology (current: Guangdong Academy of Agricultural Sciences). The *Escherichia coli* K-12 BW25113 hyper-mutator strain (Δ*mutS*) (21) was provided by Dr. Ryuichi Koga at the National Institute of Advanced Industrial Science and Technology. The *Escherichia coli* K-12 BW25113 Δ*gatC* strain of the Keio collection (53) were purchased from the National BioResource Project (Mishima, Shizuoka, Japan). To construct multiple-mutant strains, we removed the *ntpII* gene, flanked by FLP recognition sites (FRT), from the single mutant genome by transformation of pFLP3. pFLP3 was a gift from Herbert Schweizer (Addgene plasmid #64946; https://www.addgene.org/64946/; RRID:Addgene_64946). We then replaced the target gene with a kanamycin resistance gene (neomycin phosphotransferase II: *nptII*) for subsequent gene deletion by λ-Red recombination. The FRT-flanked *ntpII* genes were amplified in each mutant genome using the primers listed in Table S5. The transformants were then screened on lysogeny broth (LB) agar plates supplemented with kanamycin (25 µg/mL). To construct ∆*gatC*[cm^R^], we introduced a chloramphenicol resistance gene (chloramphenicol acetyltransferase: *cat*) into the *intS* locus. Insertion of the antibiotic genes into the target region was confirmed by colony PCR.

### Medium preparation

LB medium was prepared with 10 g/L tryptone (Becton, Dickinson and Company, Franklin Lakes, NJ, USA), 10 g/L NaCl (NACALAI TESQUE INC., Kyoto, Kyoto, Japan), and 5 g/L yeast extract (Becton, Dickinson and Company, Franklin Lakes, NJ, USA), adjusted to final volume with MilliQ water, and autoclaved. M9 + 0.4% glucose, galactose, N‐acetyl glucosamine (GlcNAc), and asparagine medium was made with M9 minimal salts, 5× (Becton, Dickinson and Company, Franklin Lakes, NJ, USA), following the manufacturer’s instructions. In brief, 56.4 g of the M9 minimal salts, 5× powder, was dissolved in 1 L of purified water and autoclaved at 121°C for 15 minutes. To prepare 1 L M9 + 0.4% carbon source media, the following components were mixed: 200 mL sterile M9 minimal salts, 5×; 778 mL sterile, purified water; 20 mL filter-sterilized solution of 20% D-(+)-glucose (NACALAI TESQUE Inc., Kyoto, Kyoto, Japan), D-(+)-galactose (FUJIFILM Wako Pure Chemical Corporation, Osaka, Osaka, Japan), N-acetyl-D-(+)-glucosamine (FUJIFILM Wako Pure Chemical Corporation, Osaka, Osaka, Japan), and L-asparagine monohydrate (Sigma-Aldrich Co. LLC, St. Louis, MO, USA); 2 mL sterile 1.0 M MgSO_4_ solution; and 0.1 mL sterile 1.0 M CaCl_2_. For the preparation of mucin-containing media (46), 0.2 M sodium dihydrogenphosphate buffer was prepared by dissolving 12 g sodium dihydrogenphosphate, Anhydrous (NACALAI TESQUE INC., Kyoto, Kyoto, Japan) in 500 mL MilliQ water, and 0.2 M disodium phosphate buffer was prepared by dissolving 14.2 g disodium hydrogen phosphate (NACALAI TESQUE INC., Kyoto, Kyoto, Japan) in 500 mL MilliQ water. Then, 0.2 M phosphate buffer (pH 7.0) was prepared by mixing 0.2 M sodium dihydrogenphosphate buffer and 0.2 M disodium phosphate buffer. A total of 2 g of mucin (Sigma-Aldrich Co. LLC, St. Louis, MO, USA), which was partially purified powder derived from the porcine stomach (bound sialic acid 0.5%–1.5%; free N-acetylneuraminic acid ≤0.2%), was dissolved per 100 mL of 10 mM phosphate buffer (pH 7.0). The mixture was autoclaved for 20 minutes at 121°C and centrifuged at 9,000 × *g* for 30 minutes at room temperature. The 60 mL of the supernatant was added to 240 mL of M9 medium.

All media were dispensed into sterile test tubes with two-position caps (Caplugs, Buffalo, NY, USA), moved to a vinyl anaerobic chamber (Coy Laboratory Products Inc., Grass Lake, MI, USA), and incubated for 24 hours to replace the medium with anaerobic conditions before the start of the experiment.

### Mutated gene screening

Three groups of wild-type BALB/c GF mice (experiment 1 [male; *n* = 3], experiment 2 [male; *n* = 2], and experiment 3 [female; *n* = 3]) were fed a high-MAC diet in independent axenic isolators. Δ*mutL* and Δ*mutS* grown at 37°C in LB medium under aerobic conditions were diluted to 5 × 10^8^ colony forming units per mL in phosphate-buffered saline (PBS). The animals in experiments 1 and 2 were gavaged with 200 µL of the Δ*mutL* suspension. The animals in experiment 3 were gavaged with 200 µL of the Δ*mutS* suspension. The mice were individually caged after inoculation. Δ*mutL*-inoculated mouse feces were collected on days 14, 28, 56, and 84. We could only sample feces of Δ*mutL* in experiment 2 on day 84 due to human error. After sampling Δ*mutS*-inoculated mouse feces on day 21, additional young BALB/c GF mice were co-housed to transfer Δ*mutS* to new mice from elder mice. The feces of newly inoculated Δ*mutS* were collected at day 84. Same as above, feces at days 21 and 84 in experiment 3 were sampled from different mouse individuals, but the microbial population was identical. All feces were stored at −80°C until use.

DNA was extracted from feces as previously described (54). Briefly, samples were incubated with 15 mg/mL lysozyme (FUJIFILM Wako Pure Chemical Corporation, Osaka, Osaka, Japan) at 37°C overnight. Then, achromopeptidase (FUJIFILM Wako Pure Chemical Corporation, Osaka, Osaka, Japan) was added to the lysates at a final concentration of 600 U/mL, and the lysates were incubated at 37°C for 8 h. After adding SDS and proteinase K (Merck KGaA, Darmstadt, Hessen, Germany) at final concentrations of 1% and 1 mg/mL, respectively, the lysates were incubated at 55°C overnight. The bacterial genomic DNA was extracted with the standard phenol/chloroform/isoamyl alcohol protocol.

After DNA extraction from fecal samples, whole genome sequencing was performed using HiSeq 2000 or NovaSeq 6000 (Illumina Inc., San Diego, CA, USA). The obtained reads were mapped against a reference sequence of *E. coli* K-12 MG1655 (NC_000913) using BWA version 0.7.5a (55), and duplicate sequences were eliminated using Picard version 1.97 (https://broadinstitute.github.io/picard/index.html). Reads were aligned by position using SAMtools mpileup version 0.1.18 (56), and variant calling and strand bias removal were performed using VarScan version 2.3.6 (57). Genes that carried an allele frequency of 20% or higher in a mouse were detected as mutated genes. Reads were then annotated by SnpEff version 3.4i (25), and differences from the reference sequence were extracted using in-house scripts. Mutations that were estimated to have “HIGH” or ”MODERATE” effects on protein functions by SnpEff version 3.4i were used in the following analysis.

In the comparison of mutated genes in mice fed different diets, we extracted mutated genes that were detected in more than half of the mice in the high-MAC diet-fed mice or low-MAC diet-fed mice, respectively (i.e., mutated genes detected in five or more mice in the high-MAC diet group, in which there were eight mice in total, and in more four or more mice in the low-MAC diet group, in which there were six mice in total).

### *In vivo* competition assays between Δ*gatC* and multiple-mutant strains

For competition between Δ*gatC* and multiple-mutant strains, 8- to 12-week-old male and female BALB/c GF mice were fed with high-MAC diet in axenic isolators. *Escherichia coli* K-12 BW25113 Δ*gatC*Δ*intS*[cm^R^], ∆*gatC*∆*araC*[km^R^], ∆*gatC*∆*malI*[km^R^], ∆*gatC*∆*melR*[km^R^], Δ*gatC*Δ*araC*Δ*malI*[km^R^], and ∆*gatC*∆*araC*∆*malI*∆*melR*[km^R^] were grown at 37°C in LB medium under aerobic conditions. They were diluted to 1 × 10^8^ CFU/mL in PBS and mixed so that the ratio of Δ*gatC*Δ*intS*[cm^R^] to mixtures of multiple-mutant strains was 1:1. The animals were gavaged with 200 µL of the suspension. Low-MAC diet-fed mice were gavaged after 1 week of diet acclimatization. We housed four mice in an isolator as a group, two mice housed in one cage, divided by sex (i.e., two sex-separated cages of two mice each, all within one isolator). Mouse feces were collected on days 1, 3, 5, 7, 10, and 14. A freshly collected fecal sample, weighing approximately 10–20 mg, was dissolved in LB liquid medium at a volume (µL) equivalent to the mass of the sample (mg) multiplied by 50. The original solution was diluted to 10^−3^, 10^−5^, and 10^−7^. Each solution was applied to 1.5% LB agar plates containing 25 µg/mL chloramphenicol for Δ*gatC*Δ*intS*[cm^R^] and 1.5% LB agar plates containing 25 µg/mL kanamycin for multiple-mutant strains at 37°C overnight under aerobic conditions. The colonies were counted for each plate. The competition index was calculated by


$$\frac{CFU\;of\;multiple-mutant\;strain}{CFU\;of\;\Delta gatC}$$

### Metabolite extraction

Metabolites in the cecal, fecal, and colon samples were extracted as previously described (58). Briefly, samples were lyophilized in the freeze dryer (TAITEC Corporation, Koshigaya, Saitama, Japan) and disrupted with a dispensing spoon. Ten-milligram samples were combined with four 3.0 mm zirconia/silica beads and 100 mg of 0.1 mm beads (TOMY SEIKO Co. Ltd., Nerima, Tokyo, Japan), and 500 µL of methanol-dissolved metabolites was extracted by vigorous shaking at 1,500 rpm for 5 minutes using the Shake Master NEO. (Bio Medical Science Co. Ltd., Shinjuku, Tokyo, Japan). The mixture was then cleaned by further shaking at 1,500 rpm for 5 minutes with 200 µL of Milli-Q water and 500 µL of chloroform containing 20 µM methionine sulfone, 20 µM 2-(N-morpholino) ethanesulfonic acid (MES) and 20 µM D-camphor-10-sulfonic acid (CSA) as internal standards for CE-TOFMS, and 200 µM ^13^C_6_ glucose as an internal standard for LC-MS/MS. The suspension was centrifuged at 4,600 × *g* for 30 minutes at 4°C in high-speed, refrigerated microcentrifuges (TOMY SEIKO Co., Ltd., Nerima, Tokyo, Japan), and 300 µL of the resulting supernatant was divided into two tubes of 150 µL each for measurement using CE-TOFMS and LC-MS/MS. The supernatant was transferred to a 5-kDa-cutoff filter column (Ultrafree MC-PLHCC 250/pk for Metabolome Analysis, Human Metabolome Technologies, Inc., Tsuruoka, Yamagata, Japan) and centrifuged at 4°C at 9,100 × *g* overnight. Then, 30 µL of the flow-through for LC-MS/MS measurement was transferred to a screw-top vial (Agilent Technologies, Inc., Santa Clara, CA, U.S.A.), and stored in a freezer at −80°C until use. The flow-through for CE-TOFMS measurement was evaporated in the CentriVap Centrifugal Vacuum Concentrator (Labconco Corporation., Kansas, MO, U.S.A.). The evaporated samples were stored at −80°C until use. The evaporated samples were dissolved in 50 µL of 200 µM trimesate and 3-aminopyrrolidine in Milli-Q water.

### Metabolome measurement using LC-MS/MS

LC-MS/MS analyses were performed as previously described (59), but with some modifications in galactitol measurement. Briefly, for galactitol measurement, a hydrophilic interaction chromatography (HILIC) polymer-based column (HILICpak VG-50 4E; 4.6 mm inner diameter [i.d.] × 250 mm length; 5 µm; Showa Denko K.K., Tokyo, Japan) was used for the separation of the sample solutions. The initial mobile phase was 93% acetonitrile and 7% Milli-Q water, and the acetonitrile gradient profile was 80%, 60%, and 93% at 30 min, 35 min, and 40 min, respectively. The flow rate was 1 mL min^−1^. For other sugar measurements, a HILIC amino column (Asahipak NH2P-50 4E; 4.6 mm i.d. × 250 mm length; 5 µm; Showa Denko K.K., Tokyo, Japan) was used for the separation of the sample solutions with guard column (Asahipak NH2P-50G 4A; 4.6 mm i.d. ×10 mm length; 5 µm; Showa Denko K.K., Tokyo, Japan). The initial mobile phase was 80% acetonitrile and 20% Milli-Q water, and the acetonitrile gradient profile was 70%, 60%, and 80% at 23 min, 35 min, and 40 min, respectively. The flow rate was 0.8 mL min^−1^.

The injection volume was 1 µL, and the duration was 40 minutes for each sample. The ion spray voltage was −4,500 V and the ion source temperature was 500°C. The peak detection and calculation of sugar concentration were performed with Analyst ver. 1.6.3 (Sciex, Framingham, MA, USA).

### Metabolome measurement using CE-TOFMS

Both positive and negative modes by CE-TOFMS were used for measuring the concentration of metabolites (60, 61). An Agilent capillary electrophoresis system (Agilent Technologies, Inc., Santa Clara, CA, USA). was used in all CE-TOFMS experiments as previously described (62). Detected peak areas were normalized with methionine sulfone for cationic metabolites and CSA for anionic metabolites. The peak detection and calculation of concentration were performed with MasterHands ver. 2.19.0.1 (63).

### *In vivo* RNA-seq of Δ*gatC* and Δ*gatC*Δ*araC*Δ*malI*

12-week-old male BALB/c GF mice (*n* = 4) were fed a High-MAC or Low-MAC diet in axenic isolators. *Escherichia coli* K-12 BW25113 Δ*gatC*[cm^R^] and ∆*gatC*∆*araC*∆*malI*[km^R^] were grown at 37°C in LB medium under aerobic conditions. They were diluted to 1 × 10^8^ CFU/mL in PBS. The animals were gavaged with 200 µL of the suspensions. After 14 days following inoculation, mice were dissected to obtain cecal contents.

RNA was extracted from the cecal contents of mice with NucleoSpin RNA Stool (Takara Bio Inc., Kusatsu, Shiga, Japan) following the manufacturer’s instructions. Then, rRNA was removed from the total RNA using Illumina Ribo-Zero Plus rRNA Depletion Kit (Illumina, Inc., San Diego, CA, USA).

RNA sequencing was performed using the Illumina NovaSeq 6000 (Illumina, Inc., San Diego, CA, USA) instrument in the paired-end 2 × 150 bp cycle mode by using NEBNext Ultra II RNA Library Prep Kit for Illumina (New England Biolabs, Inc. Ipswich, MA, USA).

The sequence generated 223,906,001 paired-end reads obtained from 12 samples. The following analysis was performed on Galaxy (https://galaxyproject.org/) (64). The quality score of the reads was visualized using FastQC (Galaxy version 0.73 + galaxy0) (65). For quality control, Trimmomatic (Galaxy version 0.38.0) (66) was performed with the following parameters: ILLUMINACLIP 2:30:10; LEADING = 20; TRAILING = 20; SLIDINGWINDOW = 4:15; MINLEN = 36. The reads that passed quality control were mapped to the *Escherichia coli* K-12 BW25113 reference genome (NZ_CP009273), provided by National Center for Biotechnology Information, using HISAT2 (Galaxy version 2.2.1+galaxy0) (67). Mapped reads were counted using featureCounts (Galaxy version 2.0.1+galaxy2) (68). Differentially expressed genes between Δ*gatC*Δ*araC*Δ*malI* and Δ*gatC* were calculated using DESeq2 (Galaxy version 2.11.40.7 + galaxy1) (42). Gene regulation was searched using RegulonDB version 10.10 (https://regulondb.ccg.unam.mx/index.jsp) (69)

### *In vitro* competition assay in M9 medium

*Escherichia coli* K-12 BW25113 Δ*gatC*[cm^R^], ∆*gatC*∆*araC*[km^R^], ∆*gatC*∆*malI*[km^R^], ∆*gatC*∆*melR*[km^R^], and ∆*gatC*∆*araC*∆*malI*[km^R^] were grown at 37°C in LB medium under anaerobic conditions in a vinyl anaerobic chamber (Coy Laboratory Products, Inc., Grass Lake, MI, US.) at 37°C with oxygen below 20 ppm. The anaerobic chamber’s gas composition was nitrogen, 10% carbon dioxide, and 4.85% hydrogen. Strains cultured in LB medium were washed with PBS twice and OD_600_ was measured with Biomate 3 (Thermo Fisher Scientific Inc., Waltham, MA, USA). The suspension was mixed so that the ratio of Δ*gatC*[cm^R^] to double or triple-mutant strains was 1:1. The mixed cultures were diluted to OD_600_ = 0.1 for M9 +0.4% mucin medium or OD_600_ = 0.01 for other M9 media. Three mL of the solution were dispensed into sterile test tubes with two-position caps (Caplugs, Buffalo, NY, USA) and incubated at 37°C under anaerobic conditions in a vinyl anaerobic chamber for 14 days. In M9 + 0.4% mucin medium, the bacterial cultures were subcultured every 48 hours into 3 mL of fresh medium at a 1/10 dilution rate. In other M9 media, the bacterial cultures were subcultured every 24 hours into 3 mL of fresh medium at a 1/100 dilution rate.

After 14 days of inoculation, the culture medium was diluted to 10^−3^, 10^−4^, and 10^−5^ with PBS. Fifty microliters of the diluted solutions was applied to 1.5% LB agar plates containing 25 µg/mL chloramphenicol for Δ*gatC*[cm^R^] and 1.5% LB agar plates containing 25 µg/mL kanamycin for double or triple-mutant strains at 37°C overnight under aerobic conditions. The colonies formed were counted for each plate. The competition index was calculated by


$$\frac{CFU\;of\;double\;or\;triple-mutant\;strain}{CFU\;of\;\Delta gatC}$$

### Statistical analysis

Statistical analyses were performed in R software version 4.1.2 (70). To compare the abundance of Δ*gatC* and multiple-mutant strains, a paired *t* test was performed with t.test function in the stats package version 4.1.2 and the *P* values were corrected with Holm’s correction for multiple comparisons with p.adjust function in the stats package. To test for differences in sugar compositions, PERMANOVA (71) was performed with adonis function in the vegan package version 2.5-7 (72). To compare the competition index of Δ*gatC*Δ*ara*Δ*malI*Δ*melR* and Δ*gatC* between high-MAC diet-fed mice and low-MAC diet-fed mice, two-way ANOVA was performed with the aov function in the stats package. To compare metabolite concentration between high-MAC diet-fed mice and low-MAC diet-fed mice, Welch’s *t* test was performed and the *P* values were corrected with Benjamini-Hochberg correction for multiple comparisons with the p.adjust function in the stats package. Adjusted *P* < 0.05 were considered statistically significant.

Schematic representation of experiments was drawn with Adobe Illustrator version 26.0.1 (Adobe Inc., San Jose, CA, USA). Plots were generated with the ggplot2 package version 3.3.5 (73), the ggVennDiagram package version 1.2.0 (74), and the ComplexHeatmap package version 2.13.1 (75) in R.

## Data Availability

Whole genome sequencing and *in vivo* RNA-seq data have been deposited in the DNA Data Bank of Japan (DDBJ) Sequence Read Archive (http://trace.ddbj.nig.ac.jp/dra/). The accession numbers for the mutated gene screening are DRA015347, DRA016043, DRA016044, and DRA016621. The accession number for *in vivo* RNA-seq is DRA015348. The original code has been deposited at https://github.com/t-tsukimi/ecoli_mutation. Any additional information required to reanalyze the data reported in this paper is available from the corresponding author upon request.

## SUPPLEMENTAL MATERIAL

Supplemental material msystems.01123-23-s0001.pdf Fig. S1 to S5. Tables S1 to S5.

ASM does not own the copyrights to Supplemental Material that may be linked to, or accessed through, an article. The authors have granted ASM a non-exclusive, world-wide license to publish the Supplemental Material files. Please contact the corresponding author directly for reuse.
