# Supplementary material for: Genetic mutation in Escherichia coli genome during adaptation to the murine intestine is optimized for the host diet
Source: mSystems. 2024 Jan 11;9(2):e01123-23. doi: 10.1128/msystems.01123-23 (PMC10878103; doi:10.1128/msystems.01123-23)
Supplement: Supplemental material — Fig. S1 to S5. Tables S1 to S5. [file msystems.01123-23-s0001.pdf]

Supplementary Materials

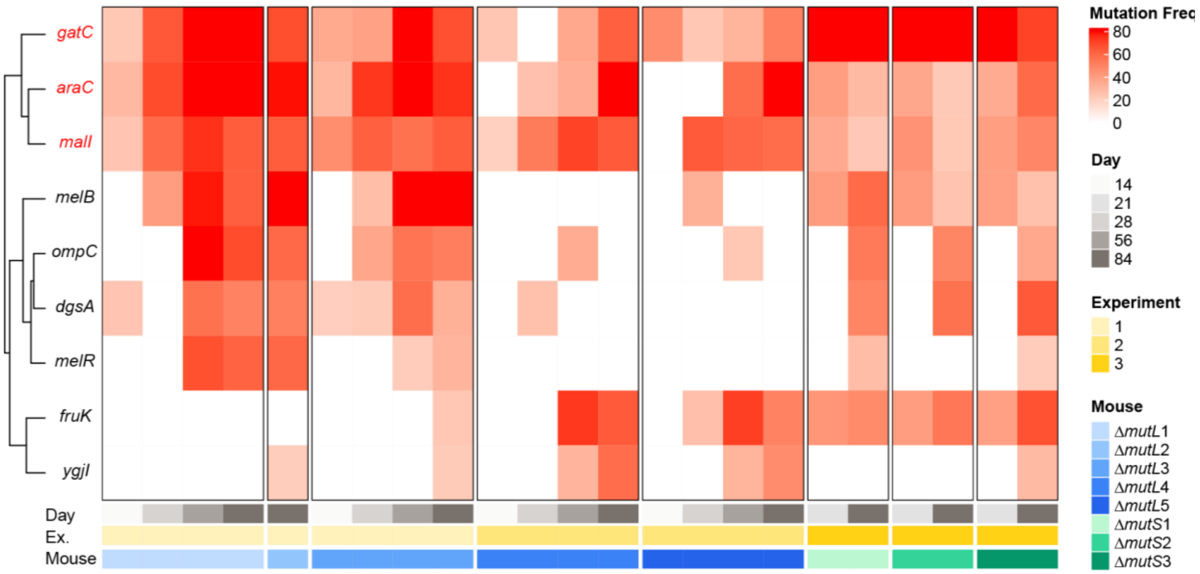

Fig. S1 Trajectories of the mutations of nine genes commonly mutated in two or more groups

Each column on the heatmap indicates a sampling timepoint in a mouse, whereas each row indicates the mutations that were predicted to have an effect on protein function. In the case where multiple-mutations were observed within a given gene, the mutation frequencies were consolidated. The dendrogram on the left represents complete clustering of Euclidean distances. The boxes on the bottom represent the sampling points of feces, experiment designation, and mouse replicates. Heatmap color indicates the mutation frequency in each mouse. Mutated genes commonly found in all mice are written in red.

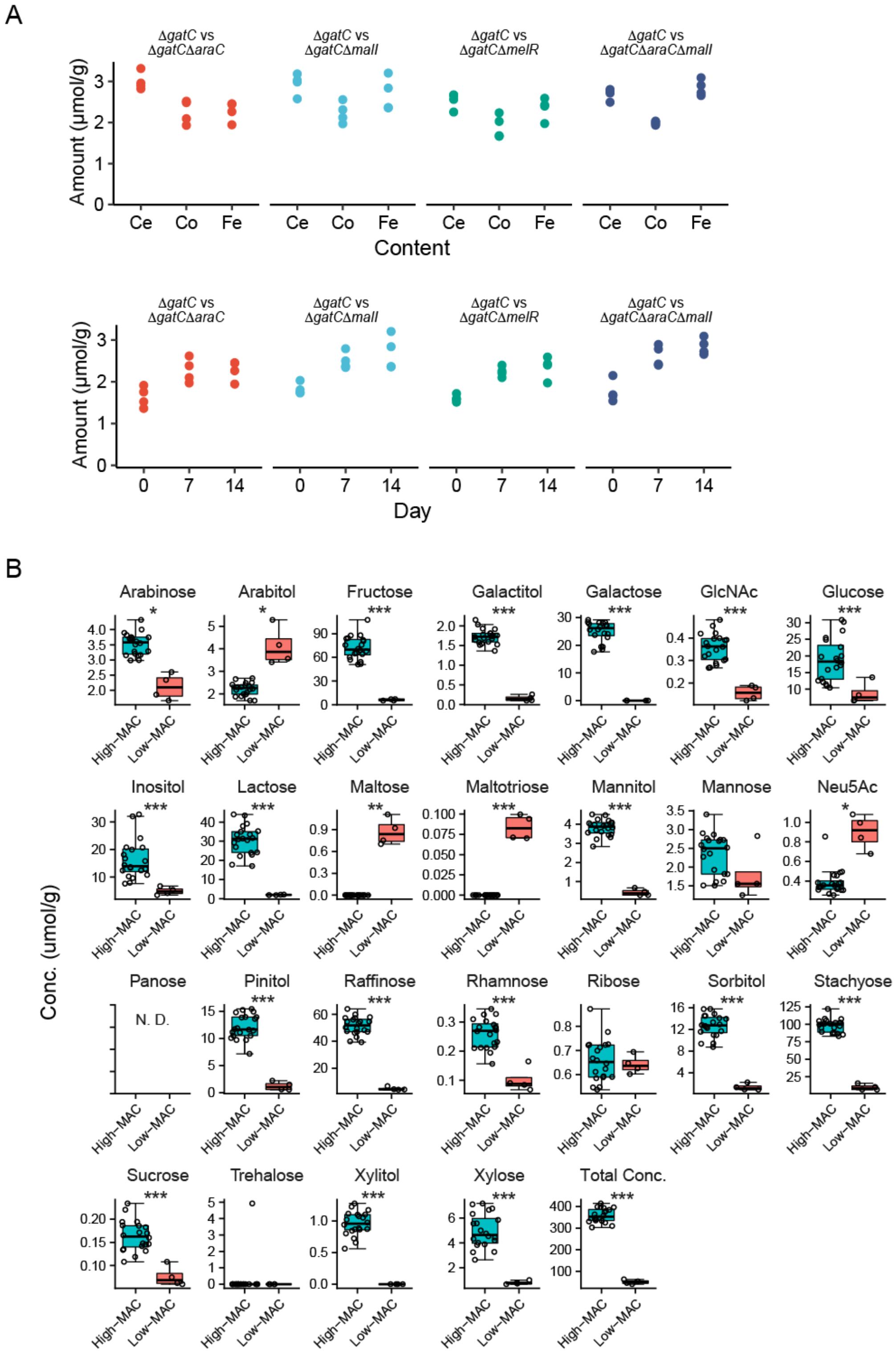

Fig. S2 The amount of galactitol in mouse intestines (A) and differences in sugar quantity in feces of high-MAC or low-MAC diet-fed mice before inoculation (B)

(**A**) The amount of galactitol in cecal contents (Ce), colonic contents (Co), and feces (Fe) on Day 14 after inoculation (top), and those in feces on Days 0, 7, and 14 (bottom). (**B**) Quantities

of sugars in feces of High-MAC or Low-MAC diet-fed mice before inoculation. *, $P < 0.05$; **, $P < 0.01$; ***, $P < 0.005$ (Welch’ s *t* test with FDR correction).

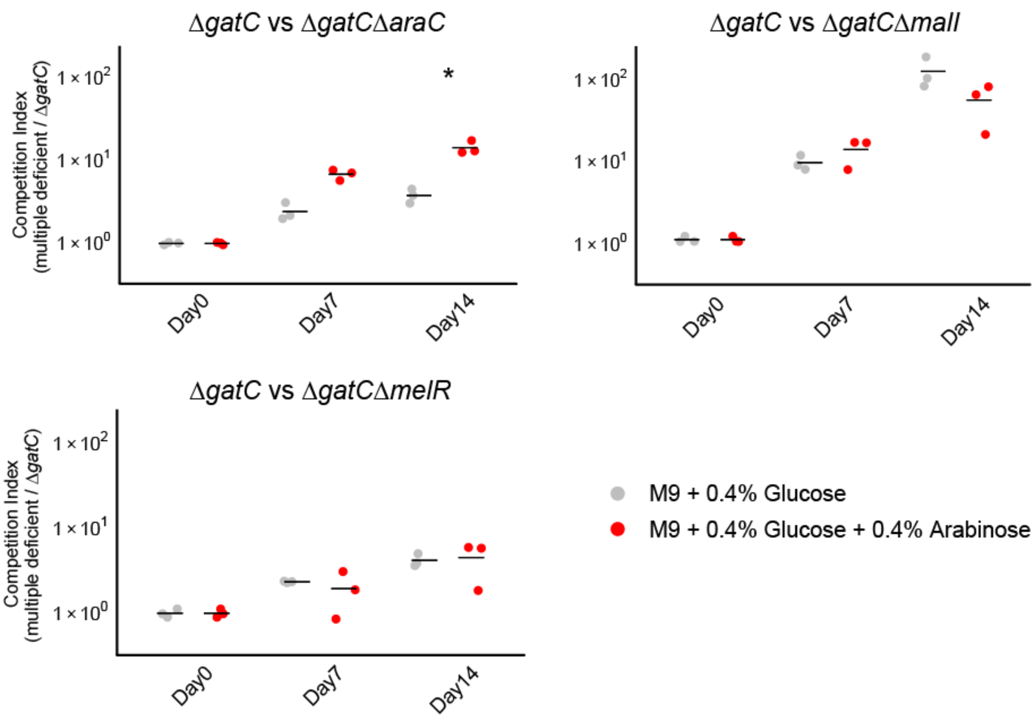

Fig. S3 Effect of arabinose on *araC*-deficient strain

In vitro competition assay between Δ*gatC* and Δ*gatC*Δ*araC* (n = 3). The log10 competition index (CFU of Δ*gatC*Δ*araC* divided that of Δ*gatC*, CI) is shown as the mean ± s.e.m. *, $P < 0.05$ (Welch's t test with Holm's correction between CI in M9 + glucose medium and that in M9 + glucose + arabinose medium on Day 14).

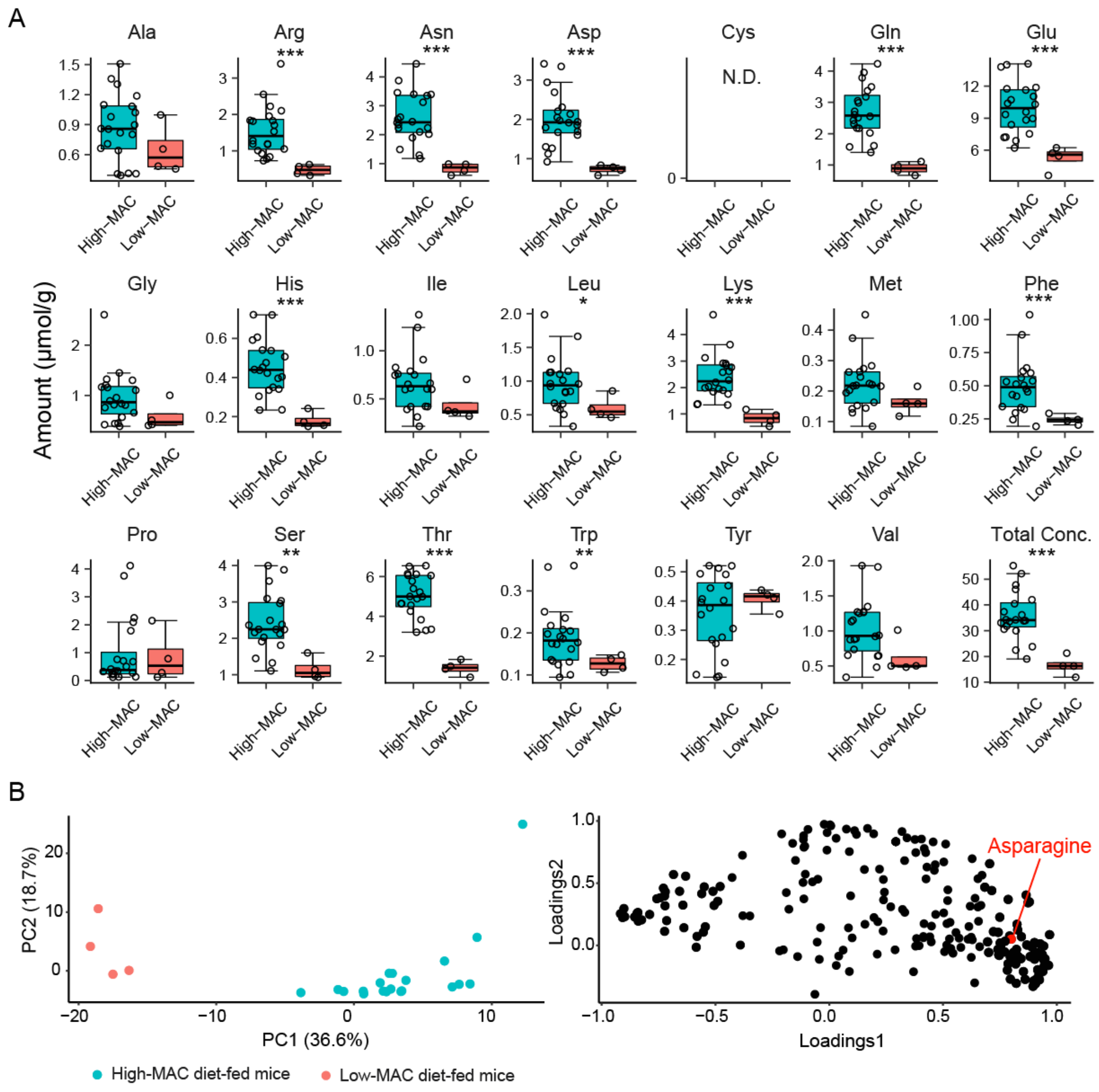

Fig. S4 Differences in quantity and composition of amino acids in mice fed different diets
(**A**) The amounts of amino acids in feces of high-MAC or low-MAC diet-fed mice before inoculation. *, $P < 0.05$; **, $P < 0.01$; ***, $P < 0.005$ (Welch's t test with FDR correction). (**B**) Principal component analysis (PCA) of metabolites detected with CE-TOFMS in feces before inoculation (left panel), and its loading plot (right panel). In the PCA panel, each point represents a sample, and the color indicates the given diet.

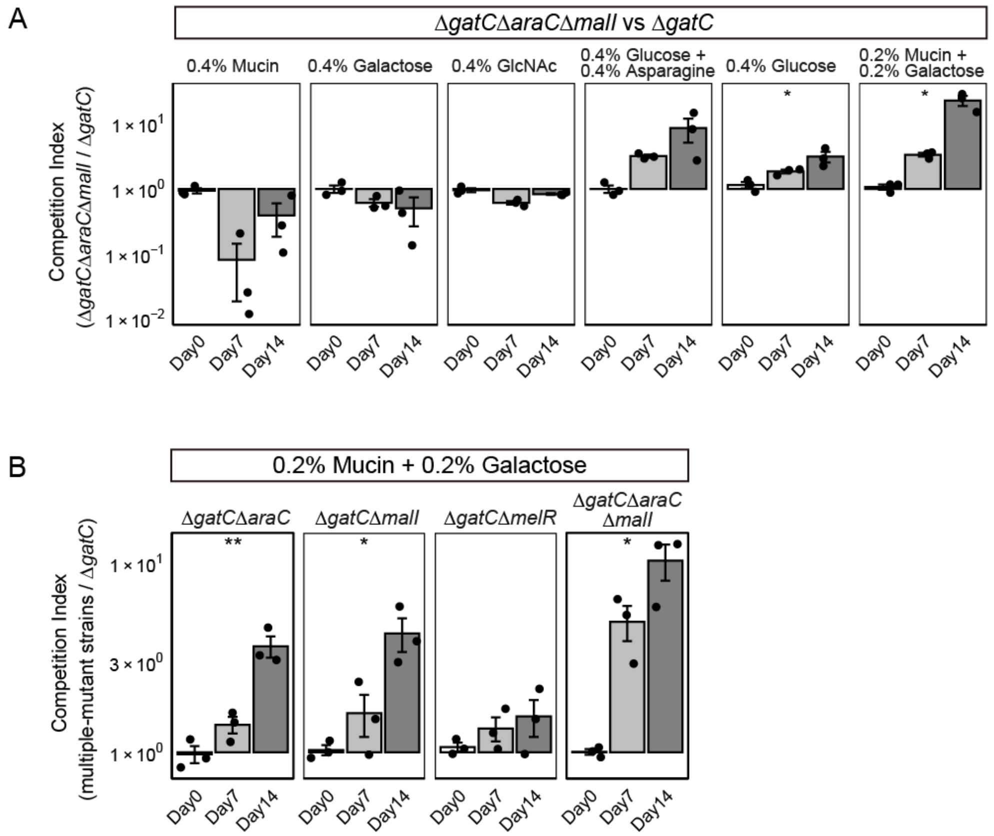

Fig. S5 *In vitro* competition assay between Δ*gatC* and Δ*gatC*Δ*araC*Δ*malI*

(A) *In vitro* competition assay between Δ*gatC* and Δ*gatC*Δ*araC*Δ*malI* in 6 media (n = 3). (B) *In vitro* competition assay between Δ*gatC* and double-mutant strains or Δ*gatC*Δ*araC*Δ*malI* in M9 + 0.2% mucin + 0.2% galactose (n = 3). The assay of Δ*gatC*Δ*araC*Δ*malI* in M9 + 0.2% mucin + 0.2% galactose medium in (A) and (B) were independently performed. The log10 competition index (CFU of a double or triple-mutant strain divided that of Δ*gatC*) is shown as the mean ± s.e.m. *, $P < 0.05$; **, $P < 0.01$ (paired *t* test with Holm's correction between Δ*gatC* and each double or triple-mutant strain on Day 14).

Table S1 Nutrient composition of high-MAC (A) and low-MAC diet (B)

A

ingredients	composition (%)
Humidity (g)	7.9
Crude protein (g)	27.8
Crude fat (g)	8.3
Crude ash (g)	6.4
Crude fiber (g)	3.1
Nitrogen-free extract (g)	46.5

B

compound	composition (%)
Milk Casein	20.0
L-Cystine	0.3
Cornstarch	39.7
Pregelatinized cornstarch	13.2
Granulated sugar	10.0
Purified soybean oil	7.0
Cellulose powder	5.0
Mineral mix (AIN-93G)[a]	3.5
Vitamin mix (AIN-93VX)[a]	1.0
Choline bitartrate	0.25
Tertiary butylhydroquinone	0.0014

[a]Vitamin and mineral mix details are provided on manufacturer's web page (see Materials and Methods).

Table S2 Commonly mutated genes detected in more than half of low-MAC diet-fed mice on Day 84

Gene	Function	Position	Ref.	Alt.	Mutation Frequency (%)[a]	Mutation Type
araC	Arabinose operon regulatory protein	70438	C	T	98.96	NON SYNONYMOUS CODING
		70816	GA	G	43.88	FRAME SHIFT
		71193	CG	C	56.41	FRAME SHIFT
cueO	Multicopper oxidase CueO	138296	A	G	100.00	NON SYNONYMOUS CODING
dgsA	DNA-binding transcriptional repressor Mlc	1665460	G	A	29.68	STOP GAINED
		1665912	G	GC	43.54	FRAME SHIFT
		1665912	GC	G	28.84	FRAME SHIFT
		1666180	T	TA	63.39	FRAME SHIFT
elaD	Protease ElaD	2380870	A	G	100.00	NON SYNONYMOUS CODING
envZ	Sensor histidine kinase EnvZ	3532702	G	A	53.03	NON SYNONYMOUS CODING
		3533544	A	G	47.03	NON SYNONYMOUS CODING
gatC	PTS system galactitol-specific EIIC component	2171418	G	A	21.99	NON SYNONYMOUS CODING
		2172029	C	T	96.31	STOP GAINED
		2172030	A	G	98.32	NON SYNONYMOUS CODING
gntR	DNA-binding transcriptional repressor GntR	3576188	C	T	48.10	NON SYNONYMOUS CODING
		3576514	G	A	83.79	NON SYNONYMOUS CODING
		3576535	G	A	55.38	NON SYNONYMOUS CODING
nupG	Nucleoside:H+ symporter NupG	3104913	T	C	100.00	NON SYNONYMOUS CODING
ssuC	Aliphatic sulfonate ABC transporter membrane subunit	993973	G	A	100.00	NON SYNONYMOUS CODING
ygfS	Putative electron transport protein YgfS	3026707	T	C	100.00	NON SYNONYMOUS CODING
yidE	Putative transport protein YidE	3862946	C	T	23.60	NON SYNONYMOUS CODING
		3863825	TC	T	91.05	FRAME SHIFT

[a]Mean frequency of mutation in *E. coli* within all mice that harbored this mutation on Day 84.

Table S3 Genes exhibiting increased (A) and decreased (B) expression in Δ*gatC*Δ*araC*Δ*malI* in High-MAC diet-fed mice

A

Gene[a]	Function	Log2FC	Δ3 Ave.	Δ*gatC* Ave.	*P*.adj	Significance	Activated by	Repressed by
malX	PTS enzyme IIBC component MalX	7.0	27413.4	148.5	2.0E-258	both	CRP	MalI
malY	Negative regulator of MalT activity/cystathionine β-lyase	5.2	13765.9	258.6	2.4E-115	both	CRP	MalI
ydeN	Putative sulfatase YdeN	4.4	5399.1	92.3	2.4E-34	both		AraC, GadX, NagC
galP	Galactose:H+ symporter	3.4	2963.2	155.1	2.5E-24	only High-MAC	CRP	GalR, GalS, NagC
ydeM	Putative anaerobic sulfatase maturation enzyme YdeM	2.9	371.5	21.7	1.6E-13	only High-MAC		AraC, GadX, NagC
nagB	Glucosamine-6-phosphate deaminase	2.9	2997.2	184.7	2.3E-13	only High-MAC	CRP	CRP, NagC
nagE	N-acetylglucosamine-specific PTS enzyme II	2.6	4173.3	362.4	1.0E-11	only High-MAC	CRP	CRP, NagC
yabI	DedA family protein YabI	2.3	714.9	109.7	1.4E-14	both		
thiQ	Thiamine ABC transporter ATP binding subunit	2.0	227.9	43.8	8.1E-09	both		SgrR
uidA	β-D-glucuronidase	1.7	630.0	164.6	1.6E-10	only High-MAC	CRP	UidR, UxuR
nagA	N-acetylglucosamine-6-phosphate deacetylase	1.7	1776.4	374.5	3.9E-05	only High-MAC	CRP, PhoP	CRP, NagC

[a]DEGs only in high-MAC diet-fed mice are bolded.

B

Gene[a]	Function	Log2FC	Δ3 Ave.	Δ*gatC* Ave.	*P*.adj	Significance	Activated by	Repressed by
araF	Arabinose ABC transporter periplasmic binding protein	-5.6	449.3	26576.2	3.2E-208	both	AraC, CRP	
araE	Arabinose:H+ symporter	-4.4	232.7	6264.6	1.2E-98	only High-MAC	AraC, CRP	
araG	arabinose ABC transporter ATP binding subunit	-4.3	274.4	7170.5	1.73E-81	both	AraC, CRP	
araD	L-ribulose-5-phosphate 4-epimerase AraD	-3.9	35.3	924.1	4.01E-37	only High-MAC	AraC, CRP	AraC
araH	Arabinose ABC transporter membrane subunit	-3.7	146.8	2186.0	3.56E-85	both	AraC, CRP	
ygeA	Amino acid racemase YgeA	-3.3	89.4	1054.0	5.44E-58	only High-MAC	AraC, CRP	
yfdH	CPS-53 (KpLE1) prophage; bactoprenol glucosyl transferase	-2.9	99.2	890.6	1.11E-31	both		

lysR	DNA-binding transcriptional dual regulator LysR	-2.6	53.6	376.8	3.62E-25	only High-MAC		LysR
BW25113_RS12270 (not annotated)		-2.5	45.8	340.8	1.12E-14	both		
malI	DNA-binding transcriptional repressor MalI	-2.4	15.1	117.7	4.22E-14	both	CRP	CRP, MalI

[a]DEGs only in high-MAC diet-fed mice are bolded.

Table S4 Detailed information on mice used in this study

Experiment	Strain	Genetic background	Age (week)	Sex[a]	Number of mice[b]	Laboratory
Mutated gene screening of Δ*mutL* inoculated mice fed High-MAC diet	BALB/c	wild-type	12	male	5	RIKEN
Mutated gene screening of Δ*mutS* inoculated mice fed High-MAC diet	BALB/c	wild-type	12	female	6	Tsukuba University
Mutated gene screening of Δ*mutL* inoculated mice fed Low-MAC diet	BALB/c	wild-type	12<br>5	male	1<br>5	RIKEN<br>Tsukuba University
In vivo competition assays between Δ*gatC* and multiple-mutant strains in High-MAC diet-fed mice	BALB/c	wild-type	8-9	male	8	Tsukuba University
	BALB/c	wild-type	8-9	female	8	
In vivo competition assays between Δ*gatC* and Δ*gatC*Δ*araC*Δ*malI*Δ*melR* in different diet-fed mice	BALB/c	wild-type	11-12	male	4	Tsukuba University
	BALB/c	wild-type	9-11	female	4	
In vivo RNA-seq of Δ*gatC* and Δ*gatC*Δ*araC*Δ*malI*	BALB/c	wild-type	12	male	12	Tsukuba University

[a]Mice used in *in vivo* competition assay were divided so that there were two males and two females per group.

[b]Five wild-type mice in mutated gene screening were used for two independent experiments, consisting of two and three mice.

Table S5 Primers used in the construction of mutant strains

Name	Sequence 5' to 3'	Reference
araC Fw	CGGGGTTACCGGTTGGGTTA	This study
araC Rv	TGTCAAATGGACGAAGCAGGGA	This study
malI Fw	AAACGTTTTATCAAATTTTAGTGAGGCA	This study
malI Rv	CACATAAACCTCCTGTGAACTTCA	This study
melR Fw	CGGCCTCATGATACTCGGAGAG	This study
melR Rv	CGGGCTGTAAAGCGCGTG	This study
intS Fw	TCGCTCCCATCCGTACCAG	This study
intS Rv	TCAAAGGAATGAAGTCTATCATCCAAGTC	This study
